# How Symptoms Change

**Published:** 1888-11

**Authors:** J. T. Kent


### HOW SYMPTOMS CHANGE.

Editors Homœopathic Physician:—For the interest of the members of the Rochester Hahnemannian Society, I desire to comment on the case related by Dr. Grant to the Society, and published in Homœopathic Physician, October, page 538, last line, where the Doctor gave Stannum. He notes that the patient came back and stated that the sputa had changed in the taste to "salty." Stannum has the salty taste as well as sweetish, and it is very common for a drug to convert one symptom into another within its own sphere in curing. If it converts a symptom into one not within its own sphere the cure will be slow or prove to be not a cure.

When a patient returns and reports symptoms worse or changed, it is proper to look to see if the new symptoms are found under the medicine taken. If they are found there the prescription is a good one and the physician may say to himself, *Sac. lac.* If the new symptoms are not found in the search into the same proving there are two conclusions to be settled by waiting:

I. The case may need another remedy.

II. If the case goes on to quick recovery it will be found that the new symptom or symptoms will some day belong to the pathogenetic symptoms.

It is well to keep a note of such symptoms, as they will some day fall into line as good symptoms to know.

The cultivation of this watchfulness leads to great accuracy in prescribing, as much will be gleaned that comes under useful knowledge. The field is a very large one, and the field of high potencies is especially a fertile one. Observing what develops in the aggravation of high potencies and the direction of symptoms is the grandest study in the materia medica.

J. T. Kent.

